# Supplementary material for: Whole‐Night Gentle Rocking Improves Sleep in Poor Sleepers With Insomnia Complaints
Source: J Sleep Res. 2026 Jun 10;35(5):e70392. doi: 10.1111/jsr.70392 (PMC13619013; doi:10.1111/jsr.70392)
Supplement: Supplementary file 1 [file JSR-35-e70392-s001.pdf]

SUPPLEMENTARY MATERIAL FOR

"Whole-night gentle rocking improves sleep in poor sleepers with insomnia complaints."

Authors:
Aurore A. Perrault [1,2,3,4]*, Nathan E. Cross[2,3,5], Thien Thanh Dang Vu[2,3], Sophie Schwartz [1,6,8], Laurence Bayer [1,7,8] *

Affiliations:
[1] Department of Neuroscience, Faculty of Medicine, University of Geneva, Geneva, Switzerland
[2] Department of Health, Kinesiology and Applied Physiology, Concordia University, Montreal, Canada
[3] Centre de Recherche de l'Institut Universitaire de Gériatrie de Montréal, CIUSSS Centre-Sud-de-l'Ile-de-Montréal, Montreal, Canada
[4] Sleep & Circadian Research Group, Woolcock Institute of Medical Research, Macquarie University, Sydney, NSW, Australia
[5] Brain and Mind Centre, School of Psychology, The University of Sydney, NSW, Australia
[6] Swiss Center for Affective Science, Campus Biotech, Geneva, Switzerland
[7] Center for Sleep Medicine, Geneva University Hospital, Geneva, Switzerland
[8] These authors contributed equally

*Corresponding authors
aurore.perrault@gmail.com; laurence.bayer@unige.ch

SUPPLEMENTAL RESULTS

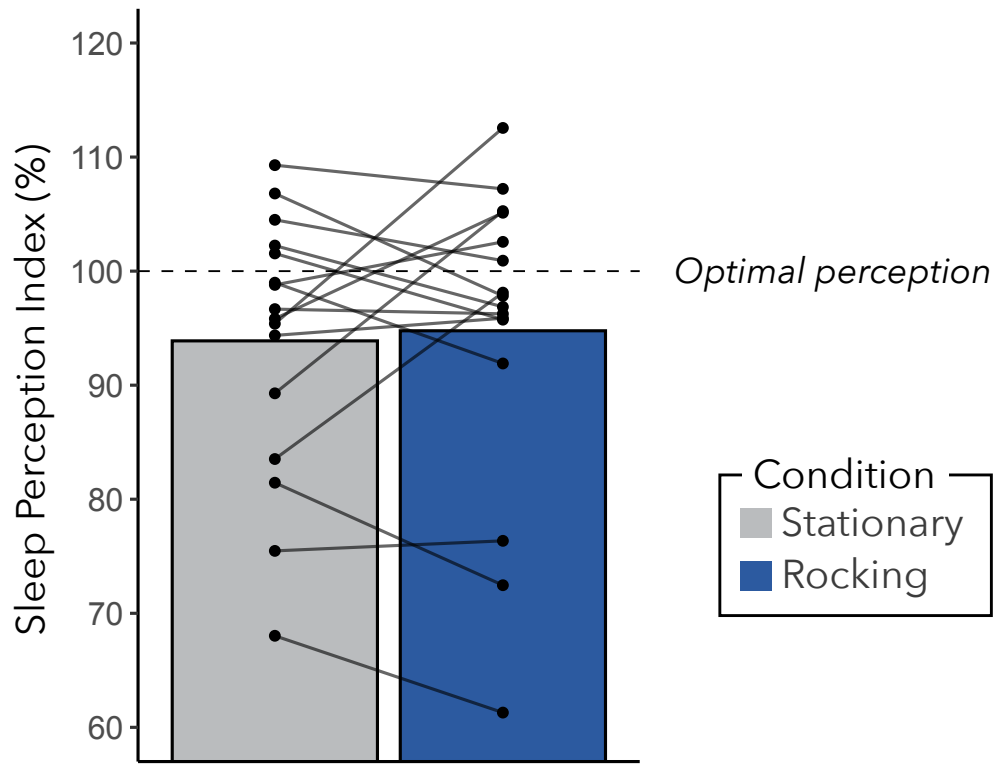

Figure S1 – Degree of sleep misperception

Mean and individual-specific sleep perception index (in%) during stationary (grey) and rocking (blue) nights (N=16)

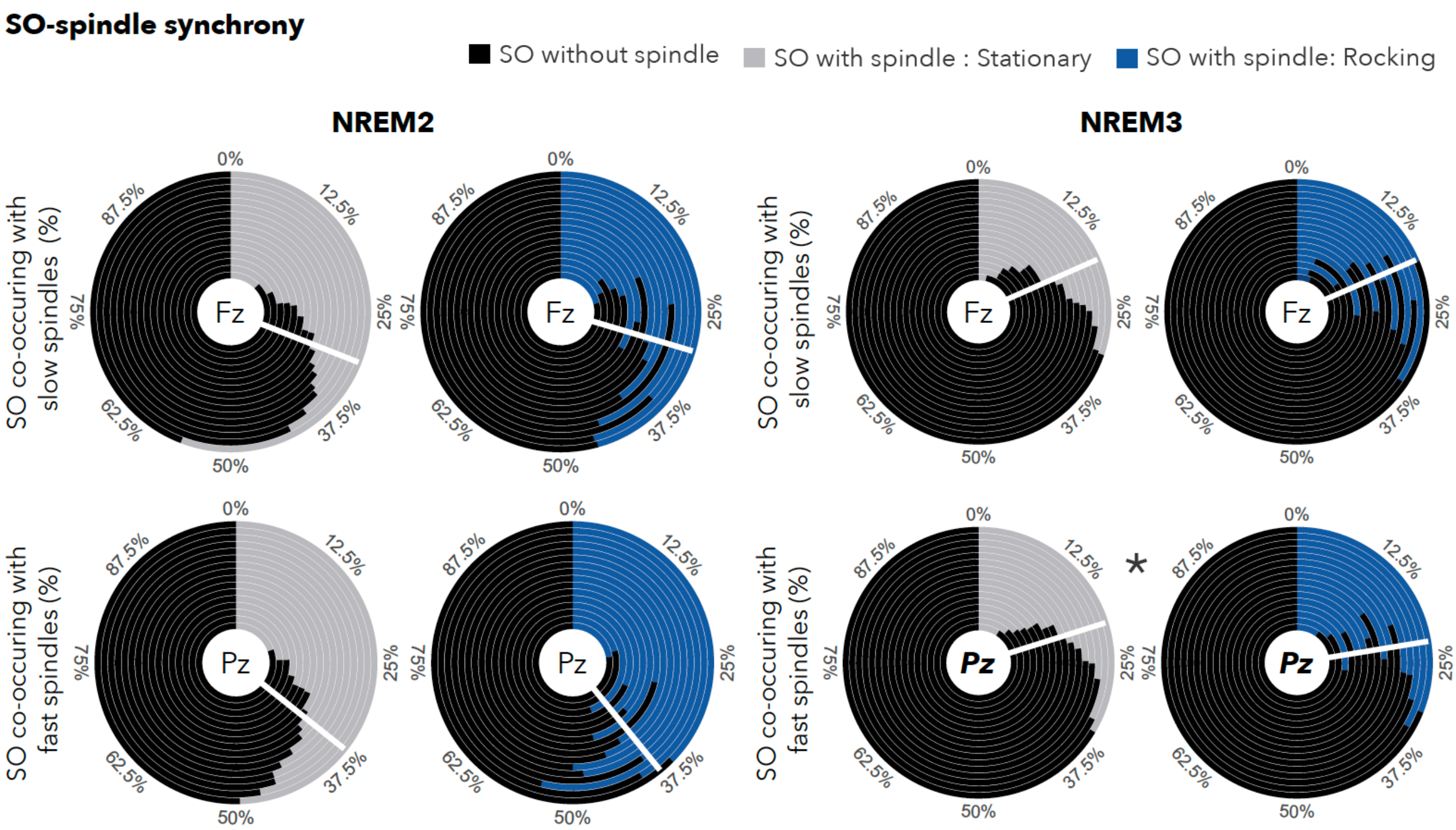

Figure S2 – SO-spindle synchrony

Racetrack plot of percentage of SO-slow spindles (detected on Fz – top) and SO-fast spindles (detected on Pz – bottom) co-occurrence (in grey/blue) and percentage of SOs without spindles co-occurring (in black) in N2 (left) and N3 (right) during stationary and rocking nights per individuals (N=16). The white bars represent the mean percentage of SO-spindle co-occurrence per night.

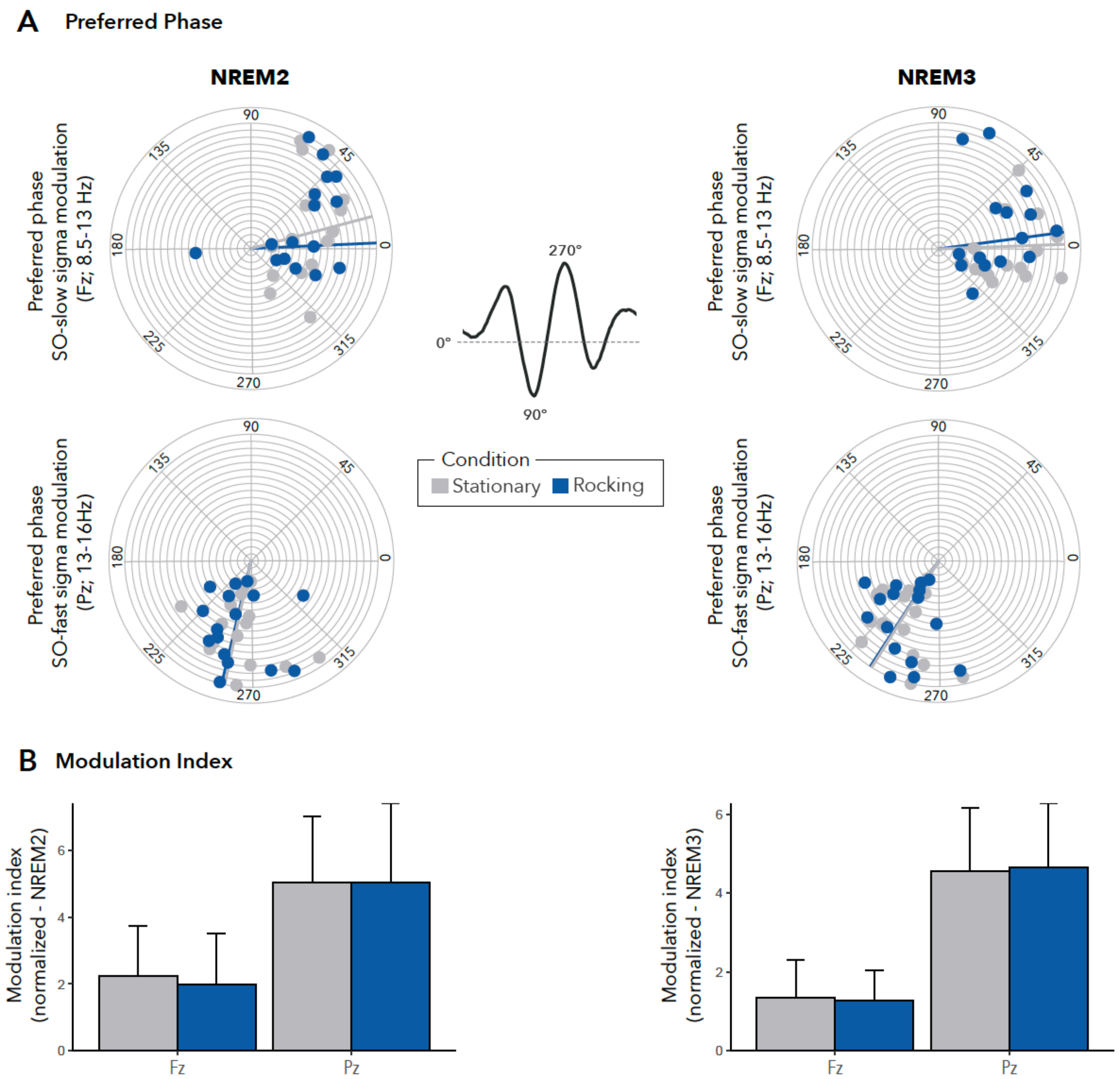

Figure S3 – No effects of rocking on phase-frequency coupling between SO and spindle activity

(A) Unit circle of preferred phase of the SO-slow sigma power modulation (Fz; top) and SO-fast sigma power (Pz; bottom) for NREM2 (left) and NREM3 (right) for each participant during stationary (grey) and rocking (blue) nights. Each line represents a participant and contains 2 small circles reflecting preferred phase during stationary and rocking nights.

(B) Mean (±SD) modulation index of SO-slow sigma power modulation (Fz) and SO-fast sigma power (Pz) for NREM2 (left) and NREM3 (right) during stationary and rocking nights

A

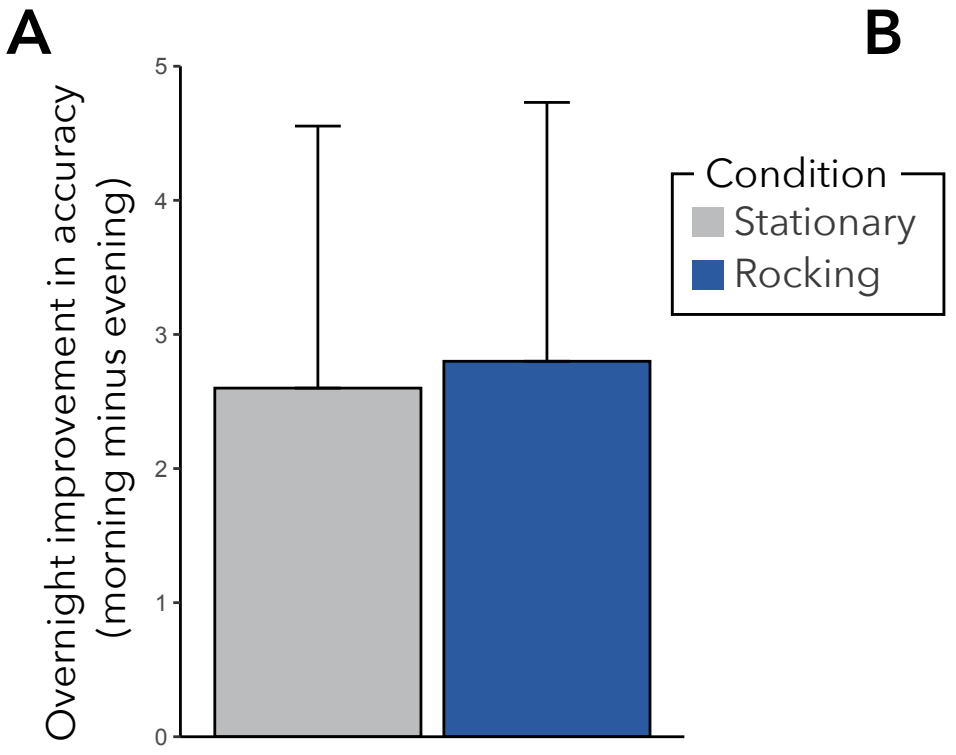

B

Stationary (mean ± sd)

	Evening	Morning	*p value*
Number of hits	14.6 ± 11.22	16.4 ± 13	*0.15*
Number of errors	9 ± 11.98	8.13 ± 11.24	*0.39*
Numbers of misses	22.33 ± 12.45	21.4 ± 13.2	*0.58*

Rocking (mean ± sd)

	Evening	Morning	*p value*
Number of hits	16 ± 11.5	17.6 ± 12	*0.19*
Number of errors	9.86 ± 9.78	8.66 ± 10.64	*0.38*
Numbers of misses	20.13 ± 11.45	19.7 ± 13.6	*0.74*

Figure S4 – No effects of rocking on overnight memory performance

(A) Mean (±SD) overnight improvement in accuracy (hits minus errors) during stationary (grey) and rocking (blue) nights.

(B) Mean (±SD) number of hits, errors and misses performed during the evening and morning recalls for stationary and rocking nights.
